# Immunomodulatory effects and mechanisms of distraction osteogenesis

**DOI:** 10.1038/s41368-021-00156-y

**Published:** 2022-01-24

**Authors:** Shude Yang, Ning Wang, Yutong Ma, Shuaichen Guo, Shu Guo, Hongchen Sun

**Affiliations:** 1 Department of Plastic Surgery, The First Hospital of China Medical University, Shenyang, China; 2 Liaoning Provincial Key Laboratory of Oral Diseases, School of Stomatology and Department of Oral Pathology, School of Stomatology, China Medical University, Shenyang, China; 3 Department of Breast Surgery, The First Hospital of China Medical University, Shenyang, China; 4 China Medical University, Shenyang, China

**Keywords:** Biomedical engineering, Bone remodelling

## Abstract

Distraction osteogenesis (DO) is widely used for bone tissue engineering technology. Immune regulations play important roles in the process of DO like other bone regeneration mechanisms. Compared with others, the immune regulation processes of DO have their distinct features. In this review, we summarized the immune-related events including changes in and effects of immune cells, immune-related cytokines, and signaling pathways at different periods in the process of DO. We aim to elucidated our understanding and unknowns about the immunomodulatory role of DO. The goal of this is to use the known knowledge to further modify existing methods of DO, and to develop novel DO strategies in our unknown areas through more detailed studies of the work we have done.

## Introduction

Distraction osteogenesis (DO) is an endogenous bone tissue engineering technology that utilizes inherent regenerative capacity to reconstruct or lengthen bone tissues.^1^ In DO, osteotomy is performed on the bone that needs to be lengthened initially. After a short latency period, a distractor is used to distract the bone ends at an appropriate speed and frequency to spontaneously promote new natural bone regeneration in the distraction gap. This period is referred to as the distraction period. Next, this process follows a long period of consolidation to achieve new bone mineralization and remodeling.^2,3^ DO is superior to other techniques used for bone defect reconstruction because this osteogenetic process does not require bone tissue transplantation.^1^ Dr. Gavriil Ilizarov, a Russian surgeon, formally proposed and popularized the DO technology in the 1950s and 1990s.^2–4^ Subsequently, its application has expanded from the limb bones to the axial and craniofacial bones. Recently, DO has been widely used in orthopedic, oral, and craniofacial and plastic surgery for congenital or acquired limb disorders, congenital knee flexion contractures, huge bone defect secondary to bone tumors, infections or trauma, craniosynostosis, Pierre Robin sequence, hemifacial microsomia, and other craniofacial dysplasias or malformations. In some diseases, DO is the preferred treatment.^4–16^ However, it should not be disregarded that a long consolidation period may lead to undesirable outcomes, such as infection at the e needle site, pain syndrome, nonunion, excessive economic burden, psychological burden, etc., which limit the clinical application of the DO^17^.

To reduce complications and improve the treatment outcomes of DO, several efforts, such as combining bone tissue engineering elements including exogenous biological scaffolds, seed cells, and growth factors with DO, have been made.^18–20^ Moreover, some studies tried to use novel targetable molecules to develop related adjuvant treatment methods in DO processes.^21^ The outcomes of these novel treatments have some disadvantages in terms of timing and duration of application.^21,22^ According to recent studies, bone tissue engineering products have immunoregulatory functions for controlling the immune microenvironment of bone regeneration sites and for reconstructing the tissue regeneration process.^23,24^ The roles of immune regulation in bone regeneration have been evaluated and used as a basis for applying bone tissue engineering technology.^25^ Although the DO is also bone regeneration and has been recognized that the immune regulations are involved in the DO, the immune microenvironment and regulation mechanisms should be explored to successfully perform DO. This review aimed to identify all studies about immunomodulatory effects during DO and summarize the changes in and effects of immune-related cytokines, signaling pathways, and cells at different periods of DO processes. Moreover, it identified some related adjuvant treatments and provided new information about immune regulation mechanisms involved in DO.

## Latency period

The latency period is similar to the initial phase of fracture. Immediately after osteotomy, hematoma forms at the osteotomy site, and inflammatory responses occur with the gathering and infiltration of different immune cells.^26^ Pro-inflammatory cytokines, such as interleukin -1(IL-1) and interleukin-6 (IL-6), are released in large quantities to debride the osteotomy site. This mechanism can provide a favorable environment for angiogenesis and osteogenesis and can promote the initial migration, proliferation, and differentiation of mesenchymal stem cells (MSCs).^26–28^ Further, to promote preliminary soft callus formation, growth factors, such as transforming growth factor-β (TGF-β), bone morphogenetic protein (BMP), insulin growth factor, and vascular endothelial growth factor (VEGF), are secreted locally by inflammatory cells and MSCs.^27,29,30^

The immunomodulation process in the latency period of DO is similar to the well-studied healing inflammatory cascade reaction process in fractures. However, there are evident differences between them.^31^ In the initial phase of fracture healing, immediate inflammatory response occurs with the higher expression of IL-1, IL-6, and tumor necrosis factor-α (TNF-α), recruitment of inflammatory cells, and promotion of extracellular matrix secretion.^32,33^ After 2–3 days, the IL-1, IL-6, and TNF-α expressions rapidly decrease to an almost undetectable level in the next endochondral phase.^28^ In contrast, only the expression of IL-1 and IL-6 increases after osteotomy during the latency period of DO. Meanwhile, that of TNF-α does not increase significantly.^28^ This phenomenon is attributed to the fact that TNF-α mainly responds to severe trauma and that osteotomy is less traumatic than fracture. However, data about chronic ethanol exposure impairing DO in a tibial DO mouse model showed that the TNF signaling pathway axis is involved.^34^ TNF receptor I-deficient mice can be free from the negative effects of chronic ethanol exposure in DO.^35^ Hence, TNF-α can be caused by other factors to participate in the DO process and affect bone regeneration.

**Fig. 1:**
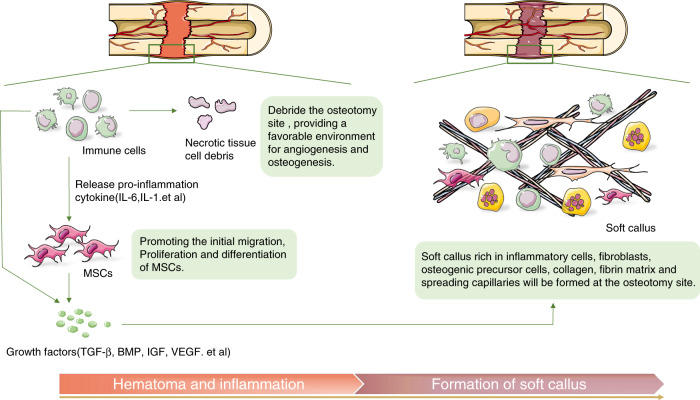
Immune-related biological processes during the latency period of DO. Hematoma is formed at the osteotomy site immediately after the osteotomy, and inflammation occurs with the aggregation and infiltration of different immune cells. Pro-inflammatory cytokines are released to debride the osteotomy site and provide a favorable environment for angiogenesis and osteogenesis. MSCs initially migrate, proliferate, and differentiate in this environment. Growth factors, such as TGF-β, BMP, etc. are secreted by local inflammatory cells and MSCs. Eventually a soft callus with inflammatory cells, fibroblasts, osteogenic precursor cells, collagen, fibrin matrix, and spreading capillaries is formed at the osteotomy site

After the inflammatory response and preliminary regeneration processes, a soft callus with inflammatory cells, fibroblasts, osteogenic precursor cells, collagen, fibrin matrix, and spreading capillaries is formed at the osteotomy site (Fig. 1).^36^ DO requires an appropriate latency period because the formation of these soft calluses is a critical step for subsequent successful bone regenerations.

## Distraction period

**Fig. 2:**
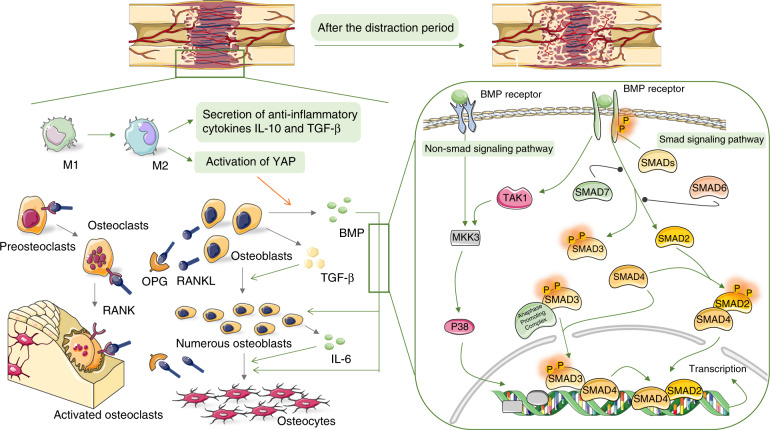
Immune-related biological processes during the distraction period of DO. Relationship between immune cells, cytokines, and inflammation-related signal pathways in the distraction period of DO. Macrophages are polarized under stretch stimulation, thereby transforming from M1 to M2 phenotype, releasing the anti-inflammatory cytokines IL-10 and TGF-β, plus TGF-β that is secreted by osteoblasts and other precursor cells together regulate immunosuppressive microenvironment and promote the proliferation of osteoblasts. Simultaneously, the activation of YAP in macrophages promotes the secretion of BMP by macrophages. Then, BMP will take over the role of TGF-β to continuallypromote the proliferation of osteogenic precursor cells, leading to an effective differentiation into osteocytes. The role of BMP is achieved via the Smad signaling pathway. Moreover, BMP can activate the MAPK kinase TAK1 to activate the MAPK-related signaling pathways, leading to a higher secretion of IL-6 in osteoblasts. IL-6 will promote the recruitment and osteogenic differentiation of osteoblast-related cells. Osteoclasts are also active during the distraction period. That is, they participate in the absorption of cartilage callus and the remodeling of new bones on both sides of the distraction gap. The expression of RANKL secreted by osteoblasts and osteocytes increases during the distraction period, and RANKL combines with RANK on osteoclast-related cells to promote the differentiation and activation of osteoclasts. Simultaneously, OPG expression is also upregulated, thereby inhibiting RANKL and regulating balance in bone resorption

The next phase is the distraction period, during which a distractor is applied to the osteotomy site to continuously stretch the osteotomy ends at an appropriate and fixed speed. Under distraction stimulation, the cartilage callus formed during the latency period is absorbed, and a surprising amount of neovascularization migrates toward the central part of the distraction gap. Moreover, several multipotent stem cells infiltrate, proliferate, and differentiate with intramembranous ossification to produce an immature woven bone, and parts of the bone at the ends of the distraction gap mineralize and remodel.^37–39^ These phenomena are caused by a series of biochemical reactions in the tissue of the gap area with a slow and stable outward traction force. Mechanochemical transduction causes changes in the microenvironment of the distraction gap,^40,41^ including migration, infiltration, and differentiation of various cells, expression of pro-inflammatory cytokines, growth factors and other biologically active substances with specific temporal and spatial characteristics, and activation of numerous inflammatory and immune-related signal pathways to regulate inflammatory responses and the immune microenvironment in the gap (Fig. 2).

### Immune cells, especially macrophage plays essential roles in the distraction period

Numerous studies have shown that an appropriate early inflammatory response during fracture healing facilitates resistance to infection and recruitment of pluripotent stem cells. Further, excessive inflammatory response interferes with new bone regeneration, revascularization, and extracellular matrix mineralization.^42–44^ The transformation from the inflammatory microenvironment to an anti-inflammatory microenvironment is necessary for successful bone regeneration, which involves phenotype changes in several immune cells, including macrophages, and T and B cells, resulting in alterations in the expression of chemokines and cytokines.^45^

Currently, it is recognized that the immunoregulation of osteogenesis is absolutely indispensable during the DO.^46^ However, whether the transformation of immune cell pro-inflammatory phenotype to anti-inflammatory phenotype occurs during the DO process remains unclear. When will it happen? What is the effect of distraction stimulation on these processes? There are only few studies discussed about these questions, and focused on the phenotypic transformation and effect of macrophages in the process of DO. Fan Zhang et al. revealed that the expression of CD68-positive macrophages increased in the distraction gap during the distraction period in a lower limb DO mouse model.^47^ When using Saporin-CD11b to induce macrophage mortality and depletion, it could decrease IL-6, BMP2, and TGF-β secretions leading to lower expressions of osteogenesis-related proteins. Hence, macrophages play an important role in DO.^47^ They identified macrophages using the CD68-positive signal, and reached the conclusion that inflammatory macrophages accelerate mechanically induced bone regeneration.^47^ Nevertheless, CD68 cannot distinguish inflammatory (M1) from anti-inflammatory (M2) macrophages. Moreover, M2 macrophages are more likely to express BMP2 and TGF-β.^48^ This finding must be further evaluated. Simultaneously, several in vitro studies showed that different mechanical stimuli had different effects on the phenotype and cytokine secretion of macrophages. However, they did not assess the role of these stimuli in the process of osteogenesis.^49–51^ A recent in vitro experiment revealed that macrophages polarize to the M2 phenotype and produce anti-inflammatory cytokines IL-10 and TGF-β to regulate the immune microenvironment under the induction of mechanical stretch.^52^ In addition, M2 can promote the osteogenic differentiation of bone marrow mescenchymal stem cells (BMSCs) under mechanical stretch stimulation via the YAP/BMP2 axis.^52^ Under stretch stimulation, a higher Wnt5a expression in the M2 activates the expression of the downstream YAP gene, which promotes the expression of BMP2 in macrophages. Then, BMP2 plays a role in BMSC osteogenesis.^52^ Moreover, Wei et al. showed the polarization of macrophages toward the M2 phenotype and the interaction between macrophages and osteogenic precursor cells in a suture DO mouse model.^53^ Results showed that chemokine C-C chemokine ligands (CCL2, CCL7, and CCL21) are produced, and they promote macrophage infiltration under stretch stimulation. Subsequently, macrophages polarize to the M2 phenotype and play an important role in promoting osteoblast differentiation of suture-derived stem cells. In addition, mechanical stretching induces macrophage polarization in vitro, which further validates that mechanical stretching has immunomodulatory effects on macrophage polarization.^53^

These studies revealed that macrophages are recruited to the distraction gap under the action of CCLs. Subsequently, macrophages undergo M2 phenotypic polarization under a specific microenvironment and stretch stimuli. M2 macrophages interact with osteogenic precursor cells and promote osteogenic differentiation by secreting BMP2, TGF-β, and other cytokines, which is similar to the immune responses of macrophages in other bone regeneration processes. Several studies have revealed that macrophages polarize to the M2 phenotype and secrete TGF-β, IL-4, BMP-2, BMP-4, IL-6, VEGF, and other cytokines to promote bone formation during regeneration.^54,55^ TGF-β and IL-4 can stimulate bone extracellular matrix secretion during bone regeneration.^56^ BMP-2 and BMP-4 promote the osteogenic differentiation of MSCs by activating the BMP and Wnt signaling pathways during fracture healing.^55^ The IL-6 family can recruit MSCs to the fracture site and induce osteoblast differentiation during fracture healing.^57^ In DO, the macrophage immune response and its interaction with osteogenic precursor cells in the process of stretch must be assessed via detailed systematic investigations. Current studies showed that M2 macrophages have potential molecular mechanisms in promoting the osteogenic differentiation of osteogenic precursor cells in the distraction gap. Further, stretch stimulation plays an important immunomodulatory effect on macrophages, which may be the major difference in macrophage immune response between DO and bone healing process. The roles of T and B cells and other immune cells in the DO process should be also identified to completely understand the whole immune regulation network in the stretching process.

### Regulatory roles of cytokines in the distraction period

IL-6, which is a pro-inflammatory cytokine, plays an important role in the inflammatory response during the latency period. Interestingly, it can be still secreted and form second lower peak by the elliptical cells, osteoblasts, and chondrocytes of the fibrous zone in rat tibial DO model until the end of the distraction period when mechanical stretch is applied.^28^ Recent studies have shown that stretch stimulation is an independent cause of IL-6 upregulation.^58^ However, IL-1 cannot be induced by stretch.^59^ Therefore, it is particularly sensitive to mechanical stretch stimulation. Function assays confirmed that IL-6 promotes osteogenic differentiation from mature osteoblast cell lines.^28^ Yuji Ando et al. revealed that IL-6 can also promote the migration and osteogenic differentiation of MSCs in a tibial DO mouse model.^46^ Therefore, it plays an important role in DO. The IL-6 expression is upregulated, thereby inducing the phosphorylation of STAT3 and, ultimately, activating osterix, an osteogenic differentiation-related transcription factor, during the process of inducing osteogenic differentiation of human adipose-derived mesenchymal stem cells.^60^ This signal axis may explain the specific mechanism of IL-6. However, whether this axis and other molecular crosstalk between the immune system and osteogenesis involved in DO was not identified.

Further, the pro-inflammatory effects of IL-6 are important.^61^ It can increase the recruitment of mononuclear immune cells in the distraction gap, and its negative effect is counteracted by the immunosuppressive effect of MSC secretions.^46^ Therefore, the immunosuppressive response is important in the distraction period. Based on clinical practice, the plasma leukocyte counts and the CRP levels are significantly elevated during the latency period in patients who received DO treatments of the tibia and fibula. Eventually, they return to normal levels during the distraction period.^62^ This finding revealed the existence of immunosuppression in the distraction phase.

TGF-β1 is another important factor of DO. Several studies showed that the expression of TGF-β1 significantly increased in the distraction period in vitro and in vivo.^59,63–67^ In addition, it is widely distributed in the distraction gap, particularly in the region of active proliferation, and cells express TGF-β receptor type II in the same location.^63–65^ Further analyses of the spatial and temporal expressions of TGF-β1 showed that TGF-β1 expression increases to more than 2-folds than that of normal control rats in a mandibular DO model. Further, TGF-β1 is mainly secreted from osteoblasts, MSCs, and connective tissues surrounding the distraction gap during the distraction period.^29^ The rabbit tibia, minipig mandibula, Chinese mountain goat tibia, sheep mandibula, and human mandibula DO models showed similar results.^68–72^ That is, stretch stimulation sustains the high expression of TGF-β1 in the distraction gap, particularly cells associated with osteogenesis. By contrast, TGF-β1 expression increased shortly after fracture healing.^68,69^ A higher TGF-β1 expression promotes osteoblast proliferation and extracellular matrix synthesis.^68,71,72^ Moreover, it is accompanied by a persistent trough in osteocalcin expression in the canine tibial distraction model. Therefore, TGF-β1 may inhibit osteoblast differentiation, decrease osteocalcin expression, and delay mineralization when TGF-β1 promotes the proliferation of osteoblasts to fill the distraction gap.^65^

Whether the number of TNF increases during the distraction phase is still debated.^27,28^ However, the expression of TNF superfamily members, NF-κB receptor-activating factor ligands (RANKL), and osteoprotegerin (OPG), which are important regulators of osteoclast activity, increased in DO.^73^ RANKL is a RANK ligand that is necessary for the differentiation and activation of osteoclasts, which can promote bone resorption.^74^ Meanwhile, OPG is expressed in osteoblasts and distributed on the bone surface beneath osteoclasts to prevent excessive bone resorption.^74^ The regulation of osteoclast activity is necessary for the bone regeneration and remodeling process of DO.^73^ In the mandibular DO rat model, the expression of RANKL steadily increased at the beginning of the distraction period.^75^ Although the expression of OPG increased and reached the peak at the end of the distraction, the RANKL-to-OPG ratio was continually increasing.^75^ After the RANKL expression became relatively dominant, the osteoclast activity increases accordingly, and the cartilage formed by natural healing during the latency period is absorbed. Moreover, bone regeneration in the gap mainly involves direct intramembranous ossification.^76^

### Signaling pathways involved in the distraction period

The focal adhesion kinase (FAK) signaling pathway is a complex, multi-pathway, multi-crossover signaling network that regulates cell migration, proliferation, and differentiation by activating intracellular PI3K/AKT, Ras/MAPK, and other inflammation-related signaling pathways in response to integrins, growth factors, and mechanical stimuli.^77^ Ransom, R. C. et al. showed that the upregulation of the FAK signaling pathway and the subsequent activation of related gene regulation programs in stem cells during the distraction period resulted in the reversal of stem cells to primitive neural crest cells in the developmental state in a mandibular DO mouse model.^78^ This is the basis of osteogenic differentiation of stem cells.^78^ Silencing the FAK signaling pathway will impair the osteogenic differentiation of BMSCs in in vitro uniaxial stretch BMSC experiments.^79^ FAK can partly achieve osteogenesis via the activation of the MAPK signaling pathway in a tibial DO rat model.^80^ Genes correlated with the FAK-MAPK signaling pathway are significantly expressed in the mid-distraction period and are maintained up to the early consolidation period. Hence, they play an important role in the process of bone regeneration.^80^

P38 is one of the most important members of the MAPK family as it controls inflammation. Moreover, it is involved in new bone regeneration during the distraction period. P38 not only increases the volume of new bones in the distraction gap by promoting the osteogenic differentiation of MSCs but also promotes the recruitment of MSCs via the stromal cell-derived factor-1/CXCR4 (SDF-1/CXCR4) axis.^81,82^ The expression of SDF-1 does not significantly increase in the early distraction period. However, it is remarkably upregulated in the late distraction period, and it reaches the peak at the end of the distraction period, which is significantly higher than that in the fracture healing zone. The high level of SDF-1 can accurately recruit more MSCs to regions with a higher BMP2 expression to promote the optimal function of BMP-2, thereby leading to sufficient new bone formation. The directional recruitment of MSCs in the distraction space by SDF-1 is indispensable for successful osteogenic differentiation.^83^ Moreover, SDF-1 is beneficial for the mineralization and remodeling of new bone by promoting the regeneration of blood vessels in the distraction space, which facilitates optimal blood flow during the consolidation phase.^82^ Coincidentally, P38 plays a key role in VEGF secretion and angiogenesis during DO.^84^ The underlying mechanism may be due to the presence of CXCR4 in endothelial progenitor cells (EPCs) as well, in parallel to the recruitment of BMSCs, which recruits a large number of EPCs.^82,85^ In addition to the SDF-1/CXCR4 axis, P38 promotes BMSC migration by controlling the secretion of matrix metalloproteinases (MMPs) that degrade the extracellular matrix in an in vitro static strain experiment of rat BMSCs.^86^ Several members of the MMP family are involved in the process of osteogenesis. MMP2 is significantly upregulated under stretch stimulation. It remarkably contributes to the osteogenic differentiation induced by stretch than other MMP members, and may have an essential role.^58,87^ Notably, MMP2 is important in the formation, proliferation, and migration of blood vessels.^88^ Enhancing the migration ability of MSCs may be one of the multiple effects of MMP2 via the P368/MMP2 pathway.^58^ The roles of all MMP family members and their interactions with other signaling molecules must be assessed in detail.

Moreover, ERK, another major member of the MAPK family, can promote the osteogenic differentiation of osteoblast precursor cells.^89^ ERK1/2 is highly expressed in the MSCs in the early distraction period and is expressed by mast chondrocytes in the late distraction period.^90^ In particular, the spatial and temporal expression of ERK1/2 is highly consistent with that of BMP2/4, and BMP can activate ERK and p38 via specific non-Smad pathways to promote osteogenic differentiation. Therefore, there is an association between the integrin-related signaling pathways and osteogenic protein expressions in the role of mechano-transduction and osteogenesis.^90,91^ However, the specific paths and mechanisms between the two remain unknown. Based on a previous study, BMP can activate transforming growth factor-β-activated kinase 1 (TAK1), a MAPK kinase kinase, which can then activate MAPK via cascade phosphorylation.^92^ In addition, the TGF superfamily activates TAK1. Then, TAK1 activates the MAPK signaling pathway to induce IL-6 expression in vitro mechanical stretching experiments on murine pre-osteoblast cell line (MC3T3-E1) and murine primary osteoblasts.^93^

Thus, the FAK signaling pathway is activated under stretch stimulation. Then, the downstream Ras/MAPK signaling pathway is activated, and it plays a role in the process of MSC recruitment and osteogenic differentiation. Activated P38 upregulates the expressions of SDF-1 and MMPs, which are migration-related signal molecules. This then leads to the recruitment of more MSCs to a specific location to assist cytokines including BMP, which play important roles in bone regeneration. In addition, SDF-1 and MMPs synergistically promote vascular regeneration, proliferation, and migration. The activated ERK is essential in the successful differentiation of osteogenic precursor cells. Its underlying mechanism may be correlated with the members of the TGF superfamily including BMP.

PIK3/AKT, another downstream pathway of FAK, also plays an important role in the DO process. Both in vivo and in vitro experiments have shown that activation of the PI3K/AKT signaling pathway can promote the differentiation of MSCs into EPCs and accelerate bone regeneration by stimulating angiogenesis in DO.^94^ Moreover, recent studies have revealed that AKT can prevent GSK-3β-mediated degradation of β-catenin by regulating the Wnt/β-catenin signaling pathway and can enhance cell viability, osteogenic differentiation, and angiogenesis gene expression of BMSCs via the AKT/GSK-3β/β-catenin axis.^95^

The Wnt signaling pathway is an immune-inflammatory related signaling pathway, and its role in bone regeneration and metabolism under mechanical stress has long been well-known.^96,97^ This pathway is continually activated during the distraction period, and it peaks at the end of this period.^80,98^ The Wnt signaling pathway is more active in the distraction period, compared with the consolidation period, and it is mainly essential in the osteogenic differentiation of osteoblast precursor cells.^80,98–100^ Further, Wnt/β-catenin participates in the differentiation of MSCs into vascular endothelial cells, which couple angiogenesis and osteogenesis.^101^ In addition, data about in vitro cyclic mechanical stretching experiments of osteogenic precursor cells showed that the non-canonical Wnt signaling pathway is involved in upregulating OPG expression to inhibit bone destruction.^102^ The expression of sclerostin, a Wnt signaling pathway inhibitor, is downregulated in long bone axial load mouse experiments. Thus, the inhibitory effect of the Wnt signaling pathway is partly relieved to promote new bone regeneration.^103,104^ Data about the use of exogenous sclerostin-antibody revealed that antagonism via the inhibitory effect of the Wnt signaling pathway can maximize the activation of the Wnt signaling pathway during the distraction period in the DO model, and can facilitate the earlier expression of osteogenesis-related proteins in BMSCs. This results in more new bone production in the distraction gap.^105,106^ These gratifying animal experiments support the potential clinical application of sclerostin-antibody in DO. Recently, the Food and Drug Administration has approved the use of Romosozumab, a sclerostin monoclonal antibody, for the treatment of osteoporosis among postmenopausal women at high risk of fractures.^107,108^ The application of sclerostin antibody in DO should be further assessed.

NF-κB, a well-known downstream signaling molecule of AKT, is a classical immune and inflammatory response-related signaling pathway in bone regeneration processes, including fracture healing, which plays a negative role.^109^ The NF-κB signaling pathway can be activated in response to stretch stimulation in mouse pre-osteoblastic cells. Moreover, it interacts with the P38 signaling pathway and jointly upregulates the expression of BMP2/4 to promote osteogenic differentiation.^110^ However, the NF-κB signaling pathway activity is downregulated during the process of mechanically stimulating the osteogenic differentiation of human jaw bone marrow mesenchymal stem cells (hJBMMSCs).^111^ The activation of the NF-κB signaling pathway via the overexpression of P65 could limit the osteogenic differentiation of these cells. Therefore, the NF-κB signaling pathway has a negative role in the process of mechanically stimulated osteogenic differentiation of MSCs.^111^ However, a recent study showed that the activity of the NF-κB signaling pathway is upregulated, and it inhibits the osteogenic differentiation of osteogenic precursor cells, osteoblast-like MG-63 cells in a mechanical stimulation experiment.^112^ Mammalian target of rapamycin (mTOR), a downstream molecule of AKT, is important for the proliferation and differentiation of osteoblasts. Simultaneously, it is significantly expressed in response to stretch stimulation. Hence, mTOR and NF-κB can work together to maintain osteogenesis at an appropriate level.^112^ These in vitro experiments have different or even contradictory results, which may be correlated with different cells, mechanical stretching methods, and selected parameters. To date, there is no study about the in vivo DO models of the NF-κB signaling.

## Consolidation period

**Fig. 3:**
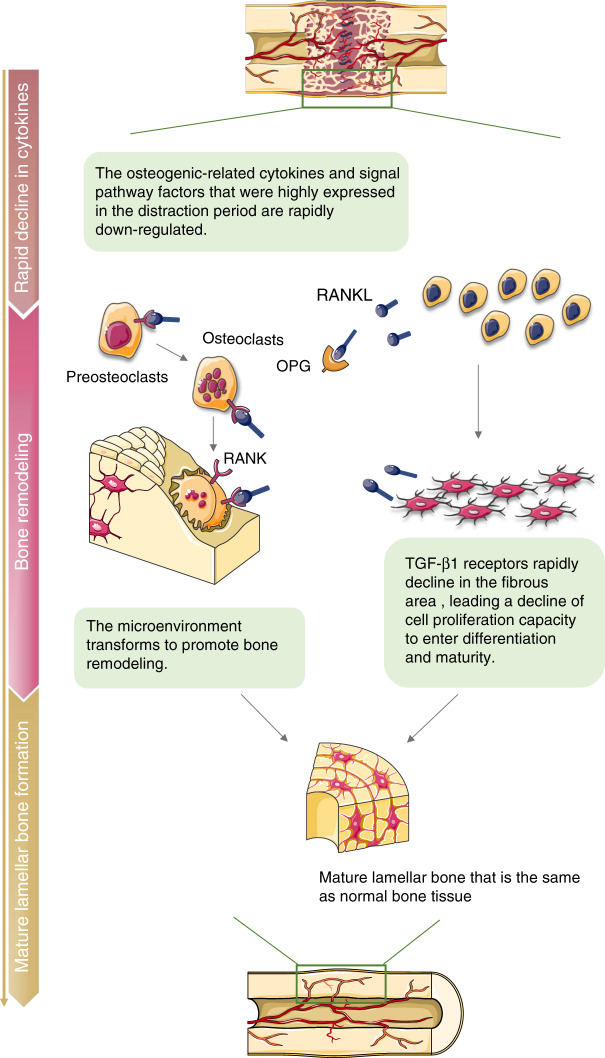
Immune-related biological processes during the consolidation period of DO. The number of osteogenic-related cytokines and signaling pathway-related molecules, which highly expressed in the distraction period, rapidly declines to achieve microenvironment transformation to promote bone remodeling. RANKL/OPG plays an important role in bone remodeling. Moreover, TGF-β1 receptors rapidly decline in the fibrous area, leading a decline of cell proliferation capacity to enter differentiation and maturity. After sufficient remodeling and mineralization, new mature lamellar bone similar to the natural bone structure are formed

After the required length of distraction has been achieved, the distraction is ceased. The new bone in the distraction gap comprises the central unmineralized zone, adjacent primary mineralized tissue zone, and peripheral remodeling braided zone.^113,114^ The following is a long period of consolidation for fusing the central unmineralized zone and completing the mineralization and subsequent remodeling of the new bone. Finally, the mature lamellar bone, which is similar to the normal structure, is generated in the distraction gap. During this period, the number of osteogenic-related cytokines and signaling pathway-related molecules, which are highly expressed in the distraction period, rapidly declines to achieve microenvironment transformation to promote bone remodeling (Fig. 3).^27,83^

TGF-β1 decreases during the consolidation phase but remains at a certain level. Further, it localizes mainly in osteoblasts and involves in the process of bone mineralization and remodeling. Then, it decreases to normal levels during the late consolidation period.^29,115^ Further, studies using several exogenous growth factors confirmed the role of TGF-β1 during the consolidation period.^116,117^ Moreover, responses to TGF-β from different cells in the distraction gap are modified due to changes in the temporal and spatial distribution of TGF-β receptors with the progression of bone maturation and mineralization.^118^ Analyses of the temporal and spatial expression of TGF-βR I (receptor for TGF-β1) in the consolidation period revealed that the receptor expression in the osteogenic area slightly decreases in the late consolidation period in a femoral DO rat model. Meanwhile, the rapid decline in the fibrous area indicates that the fibrous area loses its sensitivity to TGF-β1 prematurely, and the cell proliferation capacity decreases to enter the stage of differentiation and maturity.^118^ TGF-β receptors are classified as types I and II.^119^ TGF-β receptor type I is distributed in the bone matrix, and type II is located in the intracellular space.^72^ The spatial and temporal expressions of type II receptors, which are significantly correlated with cell proliferation, are consistent with TGF-β activation. Excessive type I will inhibit cell proliferation and promote bone mineralization.^72^ Bone volume does not significantly change in the distraction gap when applying topical exogenous TGF-β. However, the mineralization degree and bone strength increase, and they are accompanied by the downregulation of type II receptors.^72^ Due to limitations in the specific markers of type I receptor at the time, whether type I receptor is upregulated cannot be confirmed.^72^ Therefore, the temporal and spatial expressions and the effects of both TGF-β receptors in the DO process must be further investigated.

The activity of the Wnt signaling pathway remains at a certain level in the early consolidation period and decreases in the mid- and late consolidation periods.^98^ The role of Wnt in promoting bone mineralization in the process of bone healing is conclusive.^97^ Xuemei Wang et al. applied the gastric instillation of LiCL, which is an activator of the Wnt pathway in a tibia DO rat model. Results showed that the Wnt signaling pathway can increase plasm osteocalcin levels and the average mineral density and hardness of the new bone in the consolidation period. Therefore, the Wnt signaling pathway plays a role in promoting the mineralization of new bones during the consolidation period.^120^

The role of RANKL/OPG cannot be overlooked during the consolidation period. The expression of RANKL continually increases during the consolidation period, reaches its peak in the early consolidation period, and declines in the mid-consolidation period. Meanwhile, the expression of osteoprotegerin is maintained in the early and mid-consolidation period; then, it decreases.^75^ The RANKL-to-OPG ratio continually elevates during the consolidation period, reaches its peak in the late consolidation period, and then decreases at the end of the consolidation period.^75^ Osteoclasts in the bone surface and medullary cavity are active in the mid- and late consolidation periods, which are the main periods of bone resorption.^75^ Excess extra-structural new bone is resorbed, and it undergoes remodeling to form bone tissues that are similar to the normal structure.^75^ In a mandibular DO rabbit model, the expressions of IL-1β and TNF-α increase during the early consolidation period, and it is maintained until the mid- to late consolidation periods, which induces osteoclastogenesis and upregulates osteoclast activity in conjunction with RANKL.^76^ A higher RANKL-to-OPG ratio is conducive to a certain degree for bone regeneration and remodeling. However, excessive RANKL expression will likely lead to excessive bone resorption and compromise perfect bone regeneration.^121^ In a unilateral tibial DO rabbit model, the application of bovine lactoferrin can increase bone mass in the distraction gap by increasing OPG expression while slightly reducing RANKL.^122^ A recent study showed that the RANK/RANKL pathway can be upregulated by applying PTH as a bioactive substance to the distraction gap. Moreover, new bone formation and remodeling are promoted, which decreases the consolidation period in a femoral DO rat model.^123^ This may be attributed to the fact that PTH activates osteoclasts via RANKL and stimulates the production of osteoblasts, which facilitates the promotion and balance of osteoclasts and osteogenesis simultaneously.^123^

## Conclusion

DO is widely used for bone tissue engineering technology. Similar to other bone regeneration processes, immune regulation plays an important role in the DO process. However, as a unique osteogenesis method, its immune regulation mechanism has several distinct features. This study summarized immune-related events including changes in and effects of immune cells, immune-related cytokines, and signaling pathways at different periods in the DO process (Tables 1 and 2). With the use of the general bone healing process as a reference, the specific immune regulations in the process of DO were discussed. Notably, macrophage polarization, cytokine secretion, immunomodulatory roles, and crosstalk between macrophages and osteogenic precursor cells play important roles in DO. The current knowledge about immunomodulatory roles has been beneficial for DO in the current clinical practice. Nevertheless, based on this publication review, immunomodulatory mechanisms are more complex, as several molecules, different cell types, and multiple levels/steps of regulations and balances are involved to achieve optimal/effective new bone formation. There are still several unknown issues about DO. Therefore, more studies must be performed to develop novel molecular biological techniques and to optimally improve the current methods of DO.

**Table 1:** Biological processes and immune-related events in the different periods of DO

Stage of DO	Biological processes	Ref.	Immune-related events and their function	Ref.
Latency period	Formation of hematoma	^26,27,29,31,124,125^	Various immune cells infiltrate and release pro-inflammatory cytokines such as IL-1 and IL-6 to debride the osteotomy site and promote the initial migration, proliferation, and differentiation of MSCs.	^26–29,31,124–126^
	Inflammatory response			
	Formation of the outer cartilaginous callus adjacent to the periosteum and the soft callus in the gap		Inflammatory cells and MSCs release growth factors such as TGF-β, BMP, IGF, and VEGF to promote preliminary soft callus formation.	
			BMP2 and BMP4 are secreted by immature chondrocytes, and their expression can significantly decline because chondrocytes mature and secretion is ceased.	
Distraction period	Absorption of the cartilage callus	^37–39^	Immunosuppressive response plays an important role in the distraction period.	^46,62^
	A surprising amount of neovascularization and spread toward the center of the distraction gap		The expression of IL-6 appears a second lower peak in response to mechanical stretch to stimulate intramembranous osteogenesis by promoting recruitment and osteogenic differentiation of osteogenic precursor cells.	^28,46^
	Formation of fibrous interzone		TGF-β1 is continually highly expressed and is consistently distributed with type II receptors, thereby contributing to the proliferation of osteoblast precursor cells and the secretion of extracellular matrix.	^29,65,68–72^
			The expression of BMP and Smad, a downstream signaling pathway molecule, increases, thereby taking over the role of TGF-β and allowing a large number of osteogenic precursor cells to successfully differentiate into osteocytes, which play a role in both intramembranous and endochondral ossification.	^71,91,126–132^
	Multipotent stem cells infiltrate, proliferate, and differentiate with intramembranous ossification to produce immature woven bones		The RANK-to-OPG ratio continually increases, regulates the activity of osteoclasts, and promotes the absorption of cartilaginous callus that forms during the latency period.	^73–76^
	Mineralization and remodeling of parts of the bone at the ends of the distraction gap		Numerous inflammation and immune-related signal pathways, including FAK, MAPK, P38, ERK, Smad, TAK1, PIK3/AKT, Wnt, NF-κB, and mTOR respond to mechanical stimulation or cytokine signal transmission, thereby participating in angiogenesis and osteogenesis.	^78,80–82,84–86,90,93,94,98–106,109–112^
			Macrophages are widely present in the distraction gap, and M2 phenotype polarization occurs, which promotes the osteogenic differentiation of osteogenic precursor cells.	^47,52^
Consolidation period	Fusion of the central unmineralized zone	^27,83^	Osteogenic-related cytokines and signal pathway factors that were highly expressed in the distraction period are rapidly downregulated.	^27,83^
	Complete mineralization of the new bone		The expression of TGF-β1 is maintained at a certain level in the early consolidation period, and TGF-β1 participates in bone maturation and mineralization.	^29,115^
	Remodeling		The Wnt signaling pathway is maintained at a certain level in the early consolidation period, and it plays an important role in the process of bone mineralization.	^72,118^
			The RANKL-to-OPG ratio continually increases and peaks in the late consolidation period. Hence, osteoclasts, which are essential for bone remodeling, become extremely active in the mid- and late consolidation periods.	^97,98,120^
			The expression of IL-1β and TNF-α increases, and they participate in osteoclastogenesis in conjunction with RANKL.	^75,76^

**Table 2:** Changes in immune-related pro-inflammatory cytokines and growth factors during DO

	Latency period		Distraction period		Consolidation period			Ref.
Items	Early	Late	Early	Late	Early	Middle	Late	
*Pro-inflammatory cytokines*								
IL-1	↑↑↑				↑↑	↑↑	↑	^28,76^
IL-6	↑↑↑		↑↑	↑↑				^28^
TNF-α					↑↑	↑↑	↑	^28,76^
RANKL			↑↑	↑↑↑	↑↑↑	↑↑	↑↑	^73,75,76^
OPG			↑	↑↑	↑↑	↑↑	↑	^73,75,76^
RANK-to-OPG ratio			↑	↑	↑↑	↑↑↑	↑↑↑	^73,75,76^
*Growth factors*								
TGF-β1		↑↑↑	↑↑↑	↑↑↑	↑↑			^29,65,68–72,115^
BMP2 and BMP4	↑↑		↑↑↑	↑↑↑	↑↑			^71,91,126–130^
